# Molecular Research of Lipid Peroxidation and Antioxidant Enzyme Activity of *Comamonas testosteroni* Bacterial Cells under the Hexachlorobenzene Impact

**DOI:** 10.3390/ijms231911415

**Published:** 2022-09-27

**Authors:** Mariia Dimova, Andrii Tugai, Tetiana Tugai, Galyna Iutynska, Dani Dordevic, Ivan Kushkevych

**Affiliations:** 1 Zabolotny Institute of Microbiology and Virology of the National Academy of Sciences of Ukraine, Acad. Zabolotnogo Str. 154, 03143 Kyiv, Ukraine; 2 Department of Microbiology, Modern Biotechnologies, Ecology and Immunology, Institute of Biomedical Technologies, Open International University of Human Development “Ukraine”, Lvivska Str. 23, 03115 Kyiv, Ukraine; 3 Department of Plant Origin Food Sciences, Faculty of Veterinary Hygiene and Ecology, University of Veterinary Sciences Brno, Palackého tř. 1946/1, 612 42 Brno, Czech Republic; 4 Department of Experimental Biology, Faculty of Science, Masaryk University, Kamenice 753/5, 62500 Brno, Czech Republic

**Keywords:** diene and triene conjugates, Schiff bases, catalase, peroxidase, malondialdehyde, hexachlorobenzene

## Abstract

The species of *Comamonas testosteroni* is the most common human pathogen of the genus, which can be associated with acute appendicitis, infections of the bloodstream, the peritoneal cavity, cerebrospinal fluid, inflammatory bowel disease, and in general, bacteremia. According to the literature, *Comamonas testosteroni* has destructive activity to a wide range of toxic chemical compounds, including chlorobenzenes. The specified strains were isolated from the soil of the organochlorine waste landfill, where hexachlorobenzene (HCB) was predominant. These strains were expected to be capable of degrading HCB. Microbiological (bacterial enrichment and cultivating, bacterial biomass obtaining), molecular biology, biochemical (enzymatic activities, malondialdehyde measuring, peroxidation lipid products measuring), and statistical methods were carried out in this research. The reaction of both strains (UCM B-400 and UCM B-401) to the hexachlorobenzene presence differed in the content of diene and triene conjugates and malondialdehyde, as well as different catalase and peroxidase activity levels. In terms of primary peroxidation products, diene conjugates were lower, except conditions with 20 mg/L HCB, where these were higher up to two times, than the pure control. Malondialdehyde in strain B-400 cells decreased up to five times, in B-401, but increased up to two times, compared to the pure control. Schiff bases in strain B-400 cells were 2–3 times lower than the pure control. However, in B-401 cells Schiff bases under higher HCB dose were in the same level with the pure control. Catalase activity was 1.5 times higher in all experimental variants, compared to the pure control (in the strain B-401 cells), but in the B-400 strain, cells were 2 times lower, compared to the pure control. The response of the two strains to hexachlorobenzene was similar only in peroxidase activity terms, which was slightly higher compared to the pure control. The physiological response of *Comamonas testosteroni* strains to hexachlorobenzene has a typical strain reaction. The physiological response level of these strains to hexachlorobenzene confirms its tolerance, and indirectly, the ability to destroy the specified toxic compound.

## 1. Introduction

The bacterial species of *Comamonas testosteroni* are reported to be rare human pathogens. These bacteria are most often detected in the bloodstream, abdominal cavity, and cerebrospinal fluid. By reaching the human microbiome, *C. testosteroni* interacts with another bacteria, causing polymicrobial infections. There are also known cases of isolating these bacteria in the urine, the pneumonic sputum from patients with immunodeficiency, and in infected animal bite wounds [1]. These microorganisms are aerobic, Gram-negative motile bacillus, which cells are able to occur not only singly but also in pairs. Previously, *C. testosteroni* (*Pseudomonas testosteroni*) belonged to the genus *Pseudomonas* and was part of the *Pseudomonas* rRNA homology group III. Subsequently, this bacteria group was reclassified to the family *Comamonadaceae*, which includes the genus *Comamonas* with the typical species of *C. terrigena* [2]. Bacteria *C. testosteroni* has been rarely observed as an infectious agent in clinical practice. The organism has low virulence potency and infrequently causes human disease. Most of these infections are polymicrobial and these bacteria interact with other microbial communities in the host organism [1]. 

The species of *C. testosteroni* is widely found in soils, activated sludge, and seabed sediments [3]. *C. testosteroni* can degrade xenobiotic compounds (e.g., polyaromatic hydrocarbons, chlorobenzenes, pentachlorophenol) and use these toxic chemical compounds as a carbon source [3,4,5]. It has been reported that *C. testosteroni* exhibits positive chemotaxis to aromatic compounds due to sensitivity to tricarboxylic acid cycle intermediates [6]. 

Due to the destructive potential presence of *C. testosteroni* to a wide range of toxic chemical compounds, these bacteria can be seen as the factor in soil bioremediation [7,8,9]. As a result of the introduction of *C. testosteroni* into the soil contaminated with polyaromatic hydrocarbons, these bacteria interact with the resident microbiota. In this way, *C. testosteroni* strengthens the synergistic connection between the resident microorganisms and promotes the activation of microbial strains that also destruct polyaromatic hydrocarbons [10].

In the environment, hexachlorobenzene (HCB) is a toxic and persistent pollutant of air, water, soil, and biological objects (one of the 12 persistent organic pollutants is prohibited for usage by the Stockholm Convention) [11]. This substance is of public health concern because it is associated with a wide range of adverse health effects, including a probable carcinogen effect too [12]. Purifying HCB-contaminated sites by chemical methods is not only uneconomic but also environmentally dangerous since they also have a destructive effect on the fauna and flora. The search for biologically active destructors is less expensive and more ecologically safe [13]. Currently, there are studies about the ability of certain microbial groups to decompose HCB into less chlorinated compounds. The resistance mechanisms studied in microorganisms, potential HCB destructors, require special attention [14]. 

Bacteria are known to form lipid peroxidation products in cell membranes, such as diene conjugates, triene conjugates, Shiff bases, and malondialdehyde under unfavorable environmental conditions. Diene conjugates belong, for the primary and triene conjugates, to the secondary lipid peroxidation products. As a result of the addition of free radicals, fatty acid chains are broken into fragments of aldehyde groups that have high reactivity. If the break occurs on both chain sides, then a secondary product, malondialdehyde (MDA), is formed. MDA can react with NH_2_-groups of phospholipids and glucosamines, N-terminal amino acids of proteins, forming intra- and intermolecular cross links of Schiff base-types [15,16]. The MDA level depends on the lipid saturation level since peroxidation occurs only when lipids are in an unsaturated form [17], as was shown by studying *P. ananatis* cytoplasmic membrane after treatment with mesotron (herbicide). These study results indicated changes in membrane permeability, which may provide protection against herbicide cytotoxicity [18].

Ogliari et al. (2009) [19] demonstrated that the pesticide mesotrione promotes a differential activating the primary proton transport system for the enzymatic detoxification of this herbicide in maize plants, inducing changes in lipid conformation after mesotrione treatment [20]. 

Since the ability of *C. testosteroni* in destroying chlorobenzene compounds [21] is known, it is an important issue to study the reaction to hexachlorobenzene and its decomposition by these bacterial strains. The hexachlorobenzene effect on the cellular lipids remains almost unexplored, so the next stage of the work was to investigate physiological indicators characterizing the physiological cell response to the toxicant action, including the peroxidation product formation level and antioxidant protection enzymes [21]. In the literature, there are no data on the lipid peroxidation in bacteria under the hexachlorobenzene presence. Therefore, the aim of the study was to determine the lipid peroxidative products in *C. testosteroni* strains (B-400 and B-401) in response to hexachlorobenzene toxicity, as well as the catalase and peroxidase activity. The specified strains were isolated from the soil of the organochlorine waste landfill, HCB was predominant. The reason for choosing these strains is the expectation that they are capable of degrading HCB.

## 2. Results

**Figure 1:**
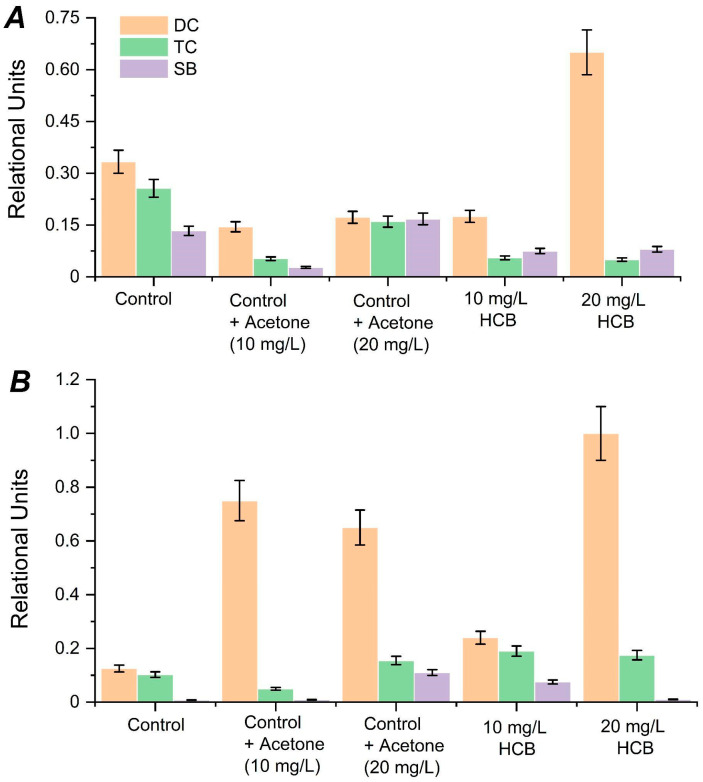
Lipid peroxidation indicators in the cells of bacterial strain B-400 (**A**) and strain B-401 (**B**) DC is diene conjugates, TC is triene conjugates, and SB is Schiff’s bases.

The study results confirmed the sensitivity of *C. testosteroni* B-400 and B-401 to the HCB influence at the physiological-biochemical level (Figure 1). An increase in the activity of lipid peroxidation (LPO) in variants with the HCB presence was noted. The *C. testosteroni* B-400 reaction to the HCB presence in the medium (Figure 1A) was characterized by the diene conjugates (DC) formation, especially at 20 mg/L doses, compared to the pure control and the acetone control required for the 20 mg/L HCB dissolution. It should be noted that the diene conjugate level was higher in the pure control than in the acetone control (for dissolving 20 mg/L HCB), which can be explained by the resistance mechanism activation, namely, an increase in the lipid saturation level [22]. At the same time, a higher Schiff base level (tertiary POL compounds) was observed (Figure 1B). Some researchers have stated that Schiff’s base formation is an adaptation mechanism since they are formed due to the bond formation between NH_2_-terminal residues of amino acids, proteins, and amino groups of phospholipids. Active transport enzymes are partially blocked, cell pores increase the MDA and DC remove from the cytoplasm, which are very toxic. Malondialdehyde is formed by the free radical interaction with the CH_3_ ends of unsaturated fatty acids; therefore, with an increase in the lipid saturation level, the MDA production decreases, as confirmed by our previous results [23].

Under the 10 mg/L HCB dose impact, at this dose, the HCB destruction level was higher, while the lipid saturation level was higher, compared to the pure control, because the cells adapted to both acetone and HCB, therefore more low indicators of POL products compared to other variants were observed (Figure 1). Under conditions of increasing HCB concentration up to 20 mg/L, a significant increase in the diene conjugates level was observed, but the secondary and tertiary LPO product level (triene conjugates and Schiff’s bases) remained approximately the same as at the lower (10 mg/L) HCB concentration. Apparently, this can be explained by the fact that in response to more toxic conditions, the synthesis of primary products (diene conjugates) increased, which induced a high activity of resistance mechanisms, that is confirmed by the catalase activity.

Physiological response of the *C. testosteroni* B-401 strain under the same cultivation conditions was different compared to the B-400 strain. It was noted that compared to the pure control, the diene conjugates level was higher in the experimental variants, namely, in the acetone controls required for dissolving 10 and 20 mL/L HCB, 20 mg/L HCB—to 6.5, 5.2, and 8.0 times, respectively. At the same time, almost the same diene and triene conjugate content was noted in the pure control. A similar reaction was noted in the variant with 10 mg/L HCB (Figure 1B). This may be related to the resistance mechanism activation, and this is why secondary LPO products were formed in significantly smaller concentrations. The tertiary product formation (Schiff’s bases) in the *C. testosteroni* B-401 cells was noted in all variants, but its highest content in the variants with acetone 20 mL/L and 10 mg/L HCB was observed. Since the Schiff base formation leads to an increase in cell pores, to make it possible to remove toxic LPO products from the cell, the DC level was the lowest from all experimental variants, and the TC level was two times higher compared to the pure control.

**Figure 2:**
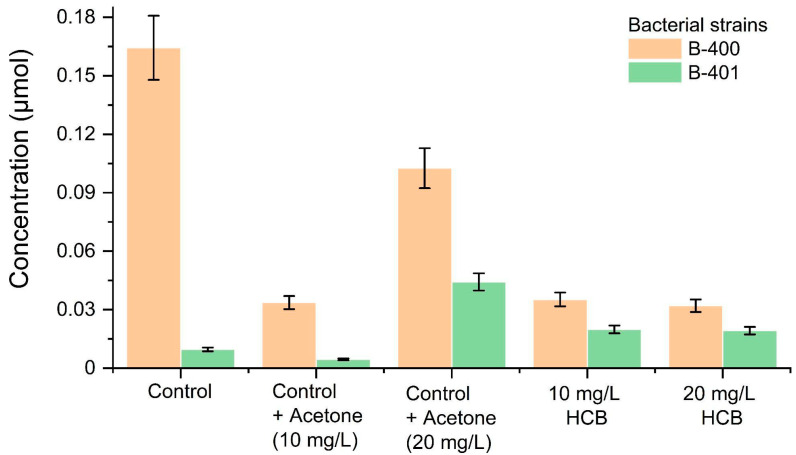
Malondialdehyde (MDA) in the cells of the bacterial strain B-400 and strain B-401.

The malondialdehyde content, as a secondary POL product, was higher in the experimental variants than in the pure control. MDA presence in the cell indicates a high probability of the formatting triene conjugates, Schiff’s bases, to cause significant disturbances in the functioning of cell membranes. Nevertheless, some researchers [24,25] believe that the MDA indicator is the last thing to pay attention during justifying the physiological response of a cell to stress, since its high level indicates the further POL formation, but with an increase in the concentration of toxic substances, MDA does not increase, and the resistance mechanisms are actively functioning (Figure 2). It should be noted that at 10 and 20 mg/L, HCB doses in the B-400 strain cells, the MDA level was lower than in the pure control, and in the B-401 strain, it was almost two times higher; however, when the dose was further increased to 20 mg/L HCB, MDA level did not increase compared to 10 mg/L HCB. Presumably, under HCB presence, the membrane saturation level in the B-400 strain significantly increased, compared to the pure control, and MDA is formed by the free radical’s interaction with the CH3-ends of unsaturated fatty acids, so MDA level decreased significantly. The MDA content in the cells from the B-401 strain did not differ at 10 and 20 mg/L HCB doses, which can also be explained by a minor increase in the saturation level of cell membranes.

Thus, the physiological reaction of the *C. testosteroni* B-400 and B-401 strains to the HCB presence in the culture medium was distinguished by a high diene conjugate content at 20 mg/L HCB doses for the first, and for the second, in all the experimental variants, relatively pure control, as well as the MDA level, which was lower in the first strain than in the pure control.

Catalase is one of the main enzymes from the antioxidant system, whose main function is to catalyze the reaction of hydrogen peroxide decomposition (to be formed as a result of biological oxidation) to water and molecular oxygen [26]. This enzyme activity in *C. testosteroni* B-400 after 7 days of cultivation was the lowest in the pure control, which was expected. At the same time, catalase activity was the highest in the variant with the acetone addition (volume for dissolving 10 mL/L HCB), to be due to the small content of diene conjugates and Schiff bases in this variant.

**Figure 3:**
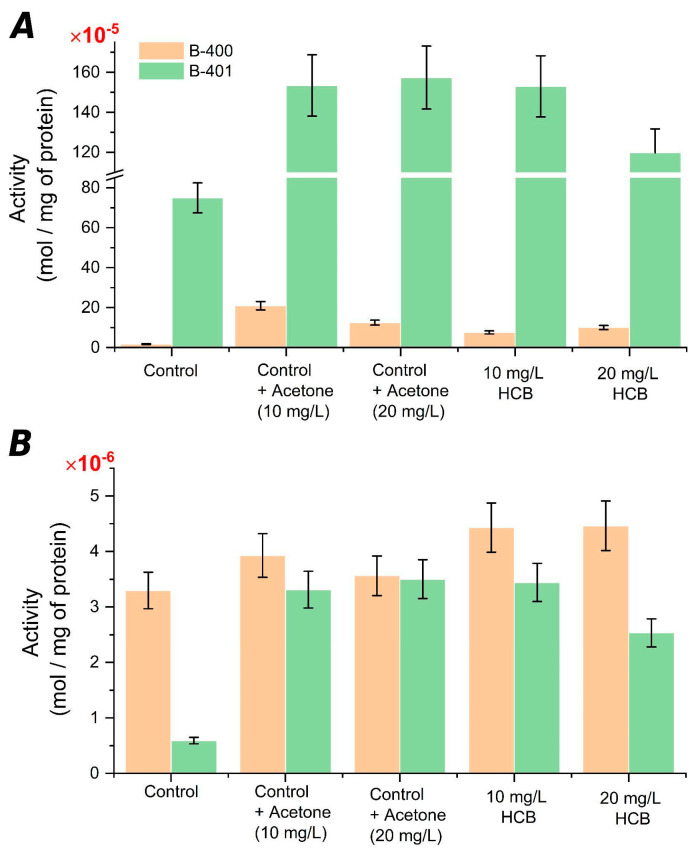
Antioxidant enzymatic activity of *Comamonas testosteroni* strain B-400 and strain B-401: catalase (**A**) and peroxidase (**B**).

Under the HCB presence, catalase activity was 3–4 times higher compared to the pure control, but lower than in controls with acetone for the corresponding concentrations; according to the results obtained, under the HCB influence, other resistance mechanisms were activated by increasing the lipid saturation level, to reduce the LPO level (Figure 3). Catalase activity in *C. testosteroni* B-401 was significantly higher than in strain B-400. In the acetone adding variants, as well as 10 mg/L HCB, catalase activity increased almost 2.5 times, compared to the pure control. Under the 20 mg/L HCB dose effect, catalase activity was slightly lower, which can be explained by a little increase in the lipid saturation level, compared to the pure control (Figure 3). Thus, catalase activity in *C. testosteroni* B-400 and B-401 strains varied depending on the strain’s sensitivity to the HCB and its dose.

An equally important enzyme of the antioxidant protection system is peroxidase since it performs the breaking down of toxic peroxides functions that are formed as a result of oxidative stress [27]. The peroxidase activity in the strain *C. testosteroni* B-400 was low, compared to the catalase activity. The activity of this enzyme was the highest (1.4 times) in cells growing in the medium containing 10 and 20 mg/L HCB than the pure control (Figure 3B). The peroxidase and catalase activity level decreasing at 20 mg/L HCB dose indicated that other resistance mechanisms were actively working under such a toxic load, due to changes in the quantitative fatty acid composition in membrane lipids.

Therefore, the study results of the antioxidant protection enzymes activity owing to the HCB effect on the *C. testosteroni* UCM B-400, B-401 cells showed that the second strain was distinguished by a higher catalase activity, while the peroxidase activity was approximately at the same level.

## 3. Discussion

Under an oxidant’s presence, the stability (mechanical strength) of biological membranes has been decreased, and antioxidant enzyme activity is able to slow down or inhibit lipid peroxidation reactions, thereby increasing membrane stability. Changes in the saturated and unsaturated fatty acid conformation of the membrane can be considered a protective bacterial mechanism that comes into contact with herbicides [28]. The decreasing unsaturated lipid content in the *E. coli* HB101 membrane after treatment with the herbicide 2,4-D was previously reported [29], and these authors considered a decrease in membrane fluidity as a possible protection mechanism against cell damage. In addition, the increasing saturation membrane lipids level of *Klebsiella planticola* DSZ was shown to make it possible to increase the cell titer in a culture medium containing the herbicide simazine [30]. In our previous studies [23], it was shown that under the toxic effect of HCB, the lipid saturation level of *Comamonas testosteroni* UCM B-400 and B-401 strains increased significantly.

Since peroxidation occurs only when lipids are in an unsaturated form, the MDA level depends on the saturation lipids level [17], the lower MDA levels in the experimental variants, compared to the control, indicate the resistance mechanism activation. For instance, a change to a higher lipid saturation level of *P. ananatis* after mesotrione treatment was shown to reduce the herbicide cytotoxicity by reducing membrane permeability [18]. 

In the literature, there is a well-founded opinion that increasing the Schiff base level is an adaptive process aimed at removing more toxic metabolites from the cells—diene conjugates and MDA. This made it possible to assume that the increment of these compound levels in different experimental variants for each *Comamonas testosteroni* strains is also an adaptive process in toxic conditions [31]. 

It should be noted that the change in the saturation level of membrane lipids was the main resistance mechanism for the B-400 strain, and the B-401 strain was characterized by a combined protective reaction: catalase activity as the predominant mechanism, and with an increasing HCB dose to 20 mg/L—an increasing membrane saturation level [23]. 

Different bacteria species are experimentally shown to use one or more enzymes of the antioxidant defense system to overcome physiological stress. For example, under oxidative stress conditions caused by a herbicides mixture (quinclorac, bensulfuron-methyl), catalase activity in *E. coli* K12 was almost at the control level, but under the same cultivation conditions in *S. maltophilia* WZ2, catalase activity increased by 50% relative to the control [32]. Establishing a MDA level was not statistically significant, in the cells of strain *Pseudomonas* sp. CMA 7.3 under the 0×, 10×, 25× working concentrations of 2,4-Dichlorophenoxyacetic acid (2,4-D), meaning that the tolerance toward herbicide was not confirmed. At the same time, catalase activity increased only under the 25× working doses of 2,4-Dichlorophenoxyacetic acid [33].

## 4. Materials and Methods

Two *Comamonas testosteroni* bacterial strains B-400 and B-401 were grown in a mineral LB medium. Sodium succinate as a carbon source (4 g/L) was added. The HCB influencing the lipid membrane properties from the studied bacteria was determined in a laboratory experiment according to the following: (a) control 1—growing the strain in LB medium (pure control), (b) control 2—growing the strain in LB medium with the addition an acetone volume required to dissolve 10 mg/L HCB, (c) control 3—growing the strain in LB with the addition the acetone volume required to dissolve 20 mg/L HCB, (d) growing the strain in LB with the addition 10 mg/L HCB, (e) growing the strain in LB medium with the addition 20 mg/L HCB. Bacteria were cultured in a liquid LB medium on a rocker (121 rpm) for 7 days at a 28 °C temperature.

After incubation, the cells were pelleted by centrifugation (20 min, 5000 rpm). Disintegrated cells were obtained by destroying sedimented cells by grinding in a mortar with liquid nitrogen.

Determination of diene conjugates was carried out by the method described in Recknagel et al. (1984) [34], adding 10 mL of hexane and isopropanol mixture (1:1) to 1 g of cell disintegrate, and the precipitate formed was removed by centrifugation (10 min, 5500 rpm). The supernatant was transferred to graduated tubes and distilled water (1:10) was added to separate the phases. After shaking twice, the upper and lower fractions were obtained and the optical density at λ = 232 nm and λ = 220 nm was determined.

To determine Schiff’s bases and triene conjugates, lipid extraction with a heptane-isopropane mixture in the ratio 3:7 was performed. A hexane and isopropanol mixture with the same ratio as a control was used [35].

Optical density (E) on a spectrophotometer (SF-26) was measured. Each phase was evaluated by comparing to the corresponding control at wavelengths: 220 nm (absorption of isolated double bonds), 232 nm (absorption of diene conjugates), 278 nm (absorption of triene conjugates), and 400 nm (absorption of Schiff bases). The content of diene conjugates, triene conjugates, and Schiff’s bases was estimated by the comparative ratios between E232/E220, E278/E220, and E400/E220, and expressed in relative units [34,36]. 

The method principle for determining malondialdehyde is MDA reacting with 2-thiobarbituric acid, forming a colored trimethyl complex at high temperature in acidic conditions with λ = 532 nm absorption maximum [36].

The optical density at 532 nm wavelength against the control was measured. The MDA concentration was calculated by the extinction coefficient according to the formula: *C* = *D*/*εl*, where *C* is the MDA concentration, μmol; *D* is optical density; *ε* is the molar extinction coefficient (1.56 × 10^5^ cm^−1^ M^−1^); *l* is the solution layer thickness in the cuvette (1 cm).

Catalase activity was determined by the spectrometric method. The conventional activity unit was obtained as the decrease in optical density in 1 mL of the reaction mixture, calculated per 1 mg of introduced protein. Catalase activity was expressed in mmoles of decomposed H_2_O_2_ min^−1^ mg^−1^ protein [37]. The protein content was determined according to Bradford’s method [38].

The amount of catalase activity in the experimental samples was calculated according to the formula:

$$A=\frac{\Delta D\times P}{\varepsilon_{\lambda }}\;\times 10^{3},$$

where: *A* is enzyme activity (mol/min/mL), Δ*D* is change in optical density per 1 min (characterized by the tangent of the inclination angle in the kinetic curve); *P* is the dilution factor; $\varepsilon_{\lambda }$ is the molar substrate extinction coefficient at the recording wavelength for hydrogen peroxide is 22.2 M^−1^ cm^−1^.

The amount of peroxidase activity was determined spectrophotometrically at 436 nm wavelength. As a substrate, 2,2′-azino-bis(3-ethylbenzothiazoline-6-sulfonic) acid (ABTS) produced by the company “Sigma” was used in an acetate buffer, pH 4.5. The reaction was started with 5 μL 3% hydrogen peroxide. Per conventional unit of activity the increase in optical density in 1 mL of the reaction mixture calculated per 1 mg of added protein was obtained [39]. The protein content was determined according to the Bradford method [38].

The amount of peroxidase activity in the test samples was calculated according to the formula:

$$A=\frac{\Delta D\times P}{\varepsilon_{\lambda }}\times 10^{3},$$

where: *A* is enzyme activity (mol/min/mL); Δ*D* is change in optical density per 1 min (characterized by the tangent of the inclination angle in the kinetic curve); *P* is the dilution factor; $\varepsilon_{\lambda }$ is the molar substrate extinction coefficient at the recording wavelength, for the ABTS is 29,500 M^−1^ cm^−1^.

## 5. Conclusions

The physiological response of *Comamonas testosteroni* to hexachlorobenzene was experimentally investigated and evaluated. A high level of diene conjugates showed a primary response to the toxicant (for strain B-400, it is higher only at doses of 20 mg/L HCB, and for strain B-401, it is higher at 10–20 mg/L HCB), the level of MDA (secondary product) at a higher dose (20 mg/L HCB) for strain B-400 was three times lower compared to the pure control, but for B-401 at the same dose, it was two times higher. Tertiary products of lipid peroxidation (Schiff bases) for the first strain were two to three times lower than in the control, and for the second, they were at the level of the control. The decrease in the level of secondary and tertiary peroxidation products under the HCB action was confirmed to be tolerated due to the catalase and peroxidase activities, as well as the saturation level of membrane lipids, which was confirmed. Thus, two main resistance mechanisms for *Comamonas testosteroni* UCM B-400 and UCM B-401 to hexachlorobenzene have been established: (i) catalase activity as a key enzyme of antioxidant protection and (ii) an increase in membrane lipid saturation. Considering the above findings, the investigated strains are promising for studying the microbial community’s destructive ability toward hexachlorobenzene.

## Data Availability

Not applicable.

## Abbreviations

HCB	hexachlorobenzene
MDA	malondialdehyde
LP	lipid peroxidation
DC	diene conjugates
TC	trine conjugates
SB	Schiff bases
LB	Luria-Bertrani medium
ABTS	2,2′-azino-bis(3-ethylbenzothiazoline-6-sulfonic) acid
